# 

**Published:** 1890-08

**Authors:** 


### CONTENTS.

*EDITORIALS.*

| | PAGE |
|---|---|
| The International Hahnemannian Association, | 337 |
| Antiseptics, | 337 |

*MISCELLANEOUS.*

| | |
|---|---|
| International Hahnemannian Association: Eleventh Annual Meeting, | 338 |
| Infant Feeding. W. I. Thayer, M. D., | 347 |
| Notes from Past Meetings of The Lippe Society, | 350 |
| Abuse of Quinine. Wm. Steinrauf, M. D., | 352 |
| A Case of Syphilis. Mahlon Preston, M. D., | 355 |
| Want to Know, You Know. Frank Kraft, M. D., | 358 |
| Fatal Errors. J. H. Jackson, M. D., | 361 |
| Hysterical Spasms Cured with Pulsatilla. Dr. Paul Lutze, | 366 |
| British Medicinal Plants. Alfred Heath, M. D., F. L. S., | 367 |
| Annual Address of George Wigg, M. D., | 370 |
| A Reply to Dr. Guernsey's Defence. Professor E. Carleton, M. D., | 377 |
| Sanicula Spring Water. J. G. Gundlach, M. D., | 378 |
| Sanicula.—A Caution. Daniel W. Clausen, M. D., | 380 |
| In Memoriam. Dr. Fred. Ehrmann, | 380 |
| Egbert Guernsey, M. D., and Ward's Island Hospital. A. McNeil, M. D., | 381 |
| Jarring. F. H. Lutze, M. D., | 382 |
| Message of Sympathy from the I. H. A. to the Venerable Dr. Wells, | 382 |

*BOOK NOTICES.*

| | |
|---|---|
| Philosophy in Homœopathy. By Chas. S. Mack, M. D., | 383 |
| Precautionary Circulars State Board of Health, | 384 |

*NOTES AND NOTICES.*

| | |
|---|---|
| Removals, | 384 |

